# Understanding the Mechanism of Action of Triazine-Phosphonate Derivatives as Flame Retardants for Cotton Fabric

**DOI:** 10.3390/molecules200611236

**Published:** 2015-06-18

**Authors:** Monique M. Nguyen, M. Sameer Al-Abdul-Wahid, Krystal R. Fontenot, Elena E. Graves, SeChin Chang, Brian D. Condon, Casey C. Grimm, Gary A. Lorigan

**Affiliations:** 1 Cotton Chemistry and Utilization Research, United States Department of Agriculture, 1100 Robert E. Lee Blvd. New Orleans, LA 70124, USA; E-Mails: krystal.fontenot@ars.usda.gov (K.R.F.); elena.graves@ars.usda.gov (E.E.G.); sechin.chang@ars.usda.gov (S.C.); brian.condon@ars.usda.gov (B.D.C.); casey.grimm@ars.usda.gov (C.C.G.); 2 Department of Chemistry and Biochemistry, Miami University, 701 E. High St. Oxford, OH 45056, USA; E-Mails: s.wahid@miamioh.edu (M.S.A.-A.-W.); lorigag@miamioh.edu (G.A.L.)

**Keywords:** triazine-phosphonate, thermal degradation, mechanism, ATR-IR, TGA-FTIR, ^31^P solid state NMR, phosphoric acid

## Abstract

Countless hours of research and studies on triazine, phosphonate, and their combination have provided insightful information into their flame retardant properties on polymeric systems. However, a limited number of studies shed light on the mechanism of flame retardancy of their combination on cotton fabrics. The purpose of this research is to gain an understanding of the thermal degradation process of two triazine-phosphonate derivatives on cotton fabric. The investigation included the preparation of diethyl 4,6-dichloro-1,3,5-triazin-2-ylphosphonate (**TPN1**) and dimethyl (4,6-dichloro-1,3,5-triazin-2-yloxy) methyl phosphonate (**TPN3**), their application on fabric materials, and the studies of their thermal degradation mechanism. The studies examined chemical components in both solid and gas phases by using attenuated total reflection infrared (ATR-IR) spectroscopy, thermogravimetric analysis coupled with Fourier transform infrared (TGA-FTIR) spectroscopy, and ^31^P solid state nuclear magnetic resonance (^31^P solid state NMR), in addition to the computational studies of bond dissociation energy (BDE). Despite a few differences in their decomposition, **TPN1** and **TPN3** produce one common major product that is believed to help reduce the flammability of the fabric.

## 1. Introduction

Phosphorus containing flame retardants (PFRs) are effective in both solid and gas phases [1,2]. In the solid phase, PFRs can increase the char yield, forming acid catalyzed char or surface barrier with inorganic glass and yield intumescences [3,4,5,6]. The formation of char is often accompanied by the release of water, which cools the surface and dilutes the combustible gases. In the gas phase, phosphorus products, such as P_2_, PO, PO_2_,may dilute the combustible polymer decomposition products (*i.e*., hydrogen atom), thereby extinguishing the fire [7,8,9]. Nitrogen containing flame retardants (NFRs) are believed to act by both mechanisms [10,11,12]. In the solid phase, they form cross-linked polymer structures that are stable at high temperatures and promote char formation. In the gas phase, NFRs releases molecules that contain nitrogen (e.g., ammonia, molecular nitrogen) which dilute the volatile polymer decomposition products [6,13]. It is well known that the combination of phosphorus and nitrogen helps to increase the performance of FRs, (*i.e*., providing higher char formation). There are many reports that show the synergistic effects between the two elements based on their ratio nitrogen/phosphorus [14,15,16]. Furthermore, the versatility of this combination in flame-retardant formulations and finishes makes it the subject of significant research studies for applications on cotton [17,18,19]. Research on flame retardants on cotton recently has extended to biomolecules and biomacromolecules which contain phosphorus and nitrogen. This new approach which combines chitosan with DNA or phytic acid provides effective anti-flammable combinations [20,21].

Most of the triazine derivatives that contain phosphorus show a spumescent property, (*i.e*., displaying foam at high temperatures). This property can be applied in intumescent coatings or claddings that produce an expanded char that acts as thermal insulation to substrates in a fire. It is a known fact that triazine-phosphonate derivatives are very effective FRs for cotton fabrics [22,23,24,25]. Although the triazine-phosphonate system technology is well developed and widely available, more studies are needed to able to fully understand the overall behavior of how this system influences the anti-flammability of treated substrates, such as cotton fabric.

**Figure 1:**
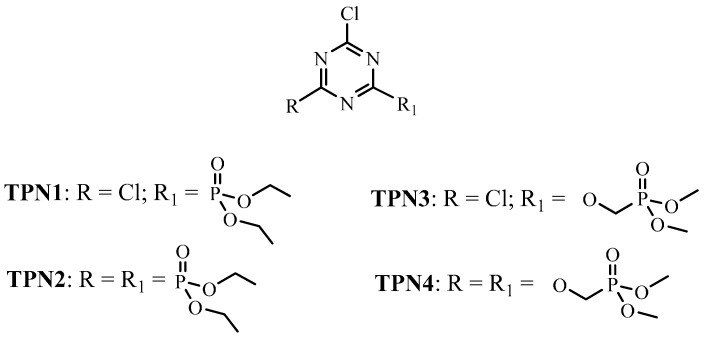
Triazine-phosphonate derivatives.

In previous reports, the authors studied the flame retardancy of four triazine-phosphorus derivatives (Figure 1) [22,23]. The four compounds were synthesized and grafted onto cotton twill fabrics to achieve different levels of add-on. All of the treated fabrics were tested for their flammability and thermal behavior against the untreated control sample. The results showed that most of the fibers in the treated samples remained intact after the flammability tests were performed. Furthermore, observations show that a swollen layer formed around them. The formation of the foam layer is directly related to the flame retardant property of the compounds since the layer could act as a barrier to prevent heat and flames from damaging the cotton fibers.

The intent of this research effort is to investigate the thermal decomposition of fabrics treated with two triazine-phosphonate derivatives diethyl 4,6-dichloro-1,3,5-triazin-2-ylphosphonate (**TPN1)** and dimethyl (4,6-dichloro-1,3,5-triazin-2-yloxy)methylphosphonate (**TPN3**) in order to gain insight into their mechanism of action. The different substituents in the structures of **TPN1** and **TPN3** offer an opportunity to elucidate structure-activity relationship. The mono-substituted flame retardants were chosen over their di-substituted analogues (**TPN2** and **TPN4**) to minimize the complications in data interpretation. To succeed in producing a protective coating for fabrics, the decomposition of the two compounds should reduce the depolymerization of cotton fabric that produces flammable gases and tars (levoglucosan) [26,27]. Furthermore, the thermal decomposition reactions are supported with the intention that they occur at lower temperatures and mainly generate char residue.

## 2. Results and Discussion

### 2.1. Fabric Treatment

During this process, the fabrics were treated with triazine-phosphonate derivatives, **TPN1** and **TPN3**, to achieve the wet pick-up values of 110 and 120 wt %, which provided the final add-on levels of 18 and 20 wt %, respectively, after curing. Samples of the **TPN1-18** and **TPN3-20** and the untreated control fabric were investigated for the mechanism of action of **TPN1** and **TPN3**.

### 2.2. Thermal Decomposition of Fabrics Treated with **TPN1** and **TPN3**: Investigation of Chemical Components in both Gas and Solid Phases

#### 2.2.1. Functional Groups Observed on the Surface of Treated Fabrics before thermal Decomposition

**Figure 2:**
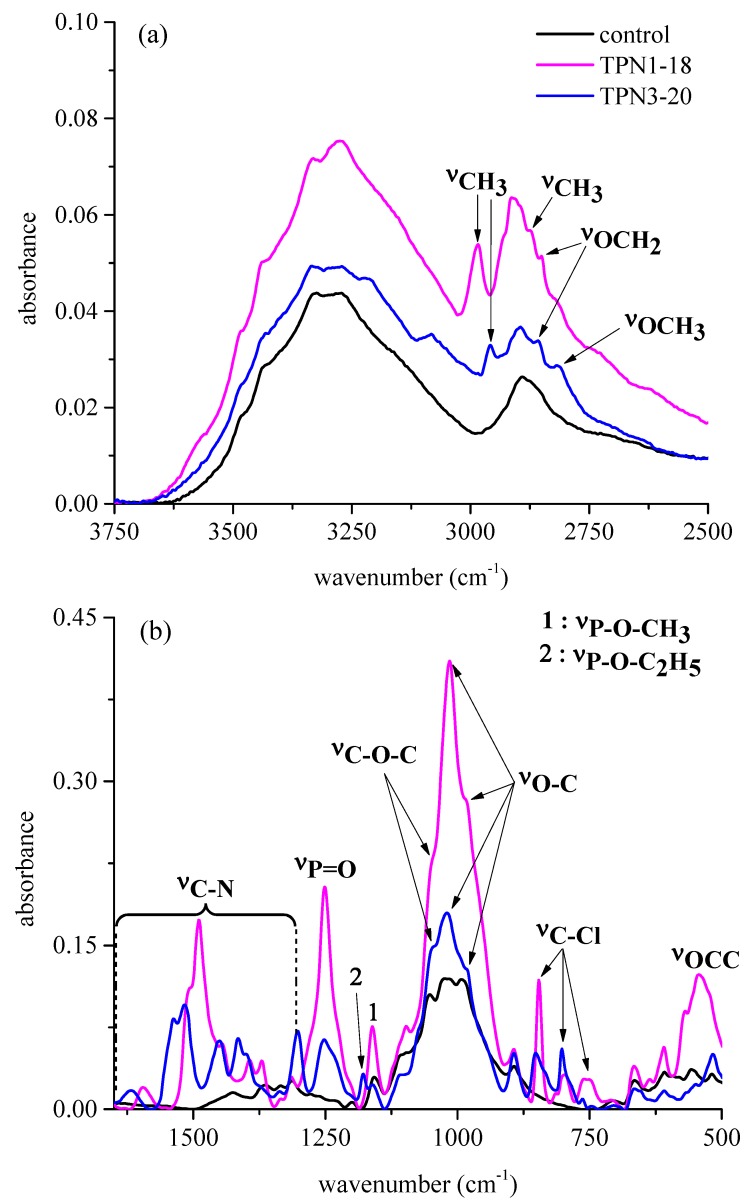
FTIR spectra of the control and treated fabrics at 3750–2500 cm^−1^ (**a**) and 1650–500 cm^−1^ (**b**).

Investigation of the functional groups on the surface of the treated fabrics before thermal degradation is necessary as this provides important details, such as the type of chemicals that are involved in the process. The results from the chemical treatment have shown significant differences between the control and the treated fabrics **TPN1-18** and **TPN3-20** (Figure 2). Although the data were collected from 4000–500 cm^−1^, the pronounced differences between the three spectra appear mostly around 3000–2750 cm^−1^ and 1650–500 cm^−1^.

At 2984 and 2958 cm^−1^ (Figure 2a), the absorptions arise from CH_3_ stretching mode of **TPN1** and **TPN3** [28]. For **TPN1**, a weak absorption at 2882 cm^−1^ also corresponds to CH_3_ stretching mode. Furthermore, both treated fabrics contain absorptions at 2851 and 2858 cm^−1^. It is reported that the characteristic absorption for CH_2_ group appears at 2853 cm^−1^ and is ascribed to the out-of-phase vibration of hydrogen atom [28]. The mean values are constant in position, *i.e.*, ±10 cm^−1^, for hydrocarbon substances including not only aliphatic compounds but also compounds such as diphenylmethane and benzyl alcohol [28]. Thus, these two absorptions belong to the CH_2_ that connects to the O (oxygen) in the two compounds. In the case of **TPN3-20**, a characteristic absorption arises at 2819 cm^−1^. This low value provides valuable group frequency for the identification of the OCH_3_ [28].

As seen in Figure 2b, the C-N triazine ring and the P=O phosphonate show their signature absorptions at 1617–1302 cm^−1^ and 1251 cm^−1^, respectively [28,29,30,31]. It is suggested that for compounds with only two electronegative substituents, such as hydrogen and alkyl phosphonates, their P=O absorption normally falls to 1275–1250 cm^−1^ [28]. At a lower region, the absorption characteristic of phosphate moieties, (e.g., the P-O-CH_3_ and P-O-C_2_H_5_) is found at 1178 and 1161 cm^−1^ [28]. Furthermore, the O-C part of the phosphonate is observed at 1020, 1015 and 980 cm^−1^ [28]. The absorptions for the P-O-CH_3_ and P-O-C_2_H_5_ groups are probably due to the rocking CH_3_ and the wagging and twisting CH_2_ [30]. The stretch at 543 cm^−1^ in **TPN1-18** is a distinctive characteristic of the OCC group. This absorption is also observed for OCC group in the HP(O)(OCH_2_CH_5_)_2_ compound [30]. These findings have confirmed the presence of the P-O-C linkage in both chemicals. In addition to these features, other stretches are also important to the identification of the two compounds. Spectroscopic features of the C-Cl appear at around 846, 797, and 760 cm^−1^ and of the C-O-C at 1060 cm^−1^. While the former corresponds to the undetached Cl on the ring [29], the latter reveals the successful anchoring of the compounds to the C6 of cellulose unit through covalent bond [28,32,33]. Previous studies indicate the presence of a stretch between 1070 and 1000 in mono-aryl ether, CH_2_-O-Ph, and this stretching frequency has to be other than *meta*-substituted aromatic ethers [28]. When the bond between the triazine ring and the cellulose unit of the fabric is formed, it does so at the *ortho-* or *para*-substituted to the nitrogen of the ring, the position of Cl. Based on these analyses, structures of both compounds remain intact during the treatment process.

#### 2.2.2. Gas Products Released during Thermal Decomposition

**Figure 3:**
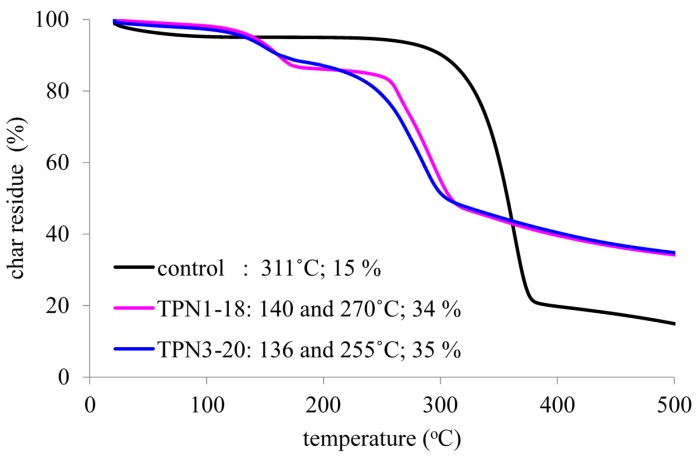
Degradation thermograms of the control and treated fabrics.

Thermal decomposition of the treated fabrics is compared to that of the control fabrics and the data are presented pictorially in Figure 3. The onset temperature and char residue of each fabric are also included. The study of degradation was performed at a temperature range of 25–500 °C for within this range the degradation activities of the FRs and cotton fabric can be easily observed. In general, the **TPN1-18** and **TPN3-20** display a lower onset of degradation in correlation to the control. Moreover, the treated fabrics exhibit a two-stage weight loss at 136 °C and 140 °C and 255 °C and 270 °C while the control shows a single-stage weight loss at 311 °C. In addition to exhibiting lower onset temperature, the treated fabrics also yield higher char at 500 °C when compared to the control. The **TPN1-18** and **TPN3-20** provide char at 34% and 35%, respectively, while the control produces char at 15%. In the two weight loss stages of the treated fabrics, the first stage loses less weight in comparison to the second stage. It is most likely that the first stage corresponds with the weight loss of the two FRs **TPN1** and **TPN3** and the second one characterizes the weight loss of the cotton fabrics. Details on both stages will be discussed below. Note that the degradation of **TPN3-20** finishes earlier than **TPN1-18** in both stages but the shape of its entire curve does not change much. This outcome is due to the insignificant weight loss in each stage of **TPN3** chemical.

**Figure 4:**
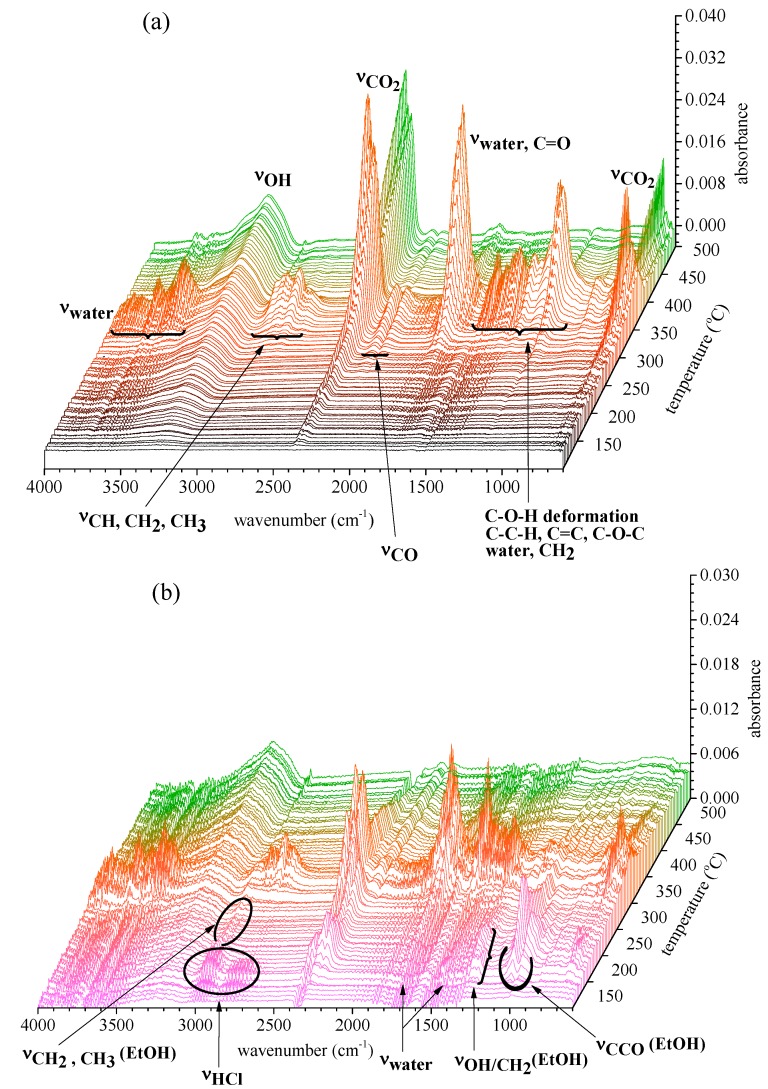

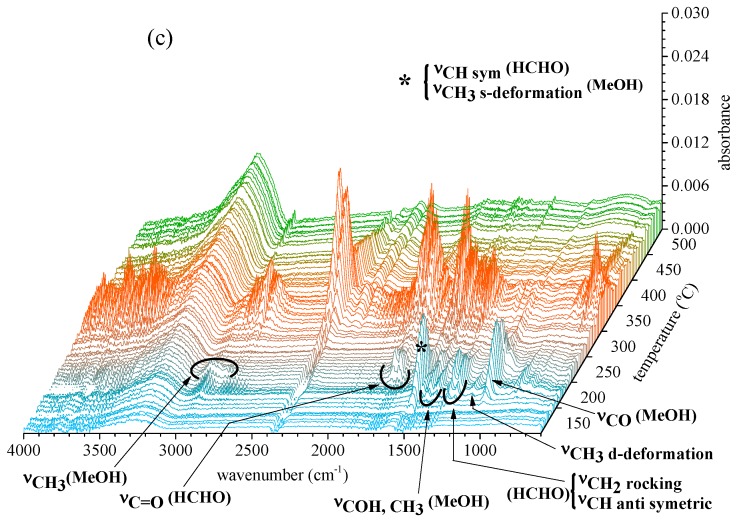
Gas profiles of the control (**a**); **TPN1-18** (**b**); and **TPN3-20** (**c**).

During the TGA process, real-time gaseous products released were detected by FTIR simultaneously. The FTIR data form a 3D spectrum, which reveals the functional groups in the gas products, which are displayed as a function of wavenumber and temperature. Typical FTIR spectra for the treated and control fabrics are shown in Figure 4.

Previous investigations on the same cotton fabric and crystalline cellulose show that most gases are products of the degradation of cellulose units [34,35]. The degradation reactions include free radicals, oxidation, dehydration, decarboxylation, decarboxylation, and decomposition of cellulose to tarry pyrolyzate-containing levoglucosan, which vaporizes and then decomposes at a later time or at a higher temperature. These products contain mainly water vapor (~4000–3500 and 1800–1500 cm^−1^); hydrocarbon OH (~3650–3500 cm^−1^); hydrocarbon CH_2_, CH_3_ (~3018–2850 cm^−1^); CO_2_ (~2380–2310 and 632–710 cm^−1^); CO (~2200–2070 cm^−1^); C=O (~1850–1600 cm^−1^) and C-O-H deformation, C-O-C, C-H, C-C and C=C (~1500–900 cm^−1^).

As shown in Figure 4, all three gas profiles exhibit three distinct stages. However, only the second stage of these three profiles shows similar features. Although these features represent the hydrocarbon OH, water vapor, CO_2_, CO, hydrocarbon C-H, C-C and C=C functional groups, the absorbance is higher for the control than the treated fabrics. A combination of the low intensity and the disappearance of absorptions between 1200 and 1000 cm^−1^ in Figure 4b,c reveals a reduction in the degradation of the treated fabrics. The above information confirms the origin of the evolved gases observed for the second stage of the treated fabrics, which arises from the degradation of the cotton fabrics. These data also reinforce the findings of the second stage in the TGA experiment.

When examining the profiles of **TPN1-18** and **TPN3-20**, one will find more similarities in their temperature dependence behaviors. In the initial stage, both hold distinctive absorptions that do not belong to the fabric. It is apparent that the released gases come from the degradation of other additives, such as the flame-retardants **TPN1** and **TPN3**. In the first stage, the two treated fabrics hold two similar characteristics: H_2_O (at 1900–1670 cm^−1^ and at 1550–1300 cm^−1^) and HCl (at 3030–2900 cm^−1^ and at 2850–2700 cm^−1^) [36].

In addition to their similarities, **TPN3-20** has some signature characteristics of its own. The absorptions at 1456, 1402, 1170 and 1054 cm^−1^ correspond to the stretches of MeOH, such as CH_3_ d-deformation and the in phase COH bend/CH_3_ rock [37,38,39]. What also manifest in this gas phase spectrum are the absorptions at 1750–1710 and 1301–1263 cm^−1^. These are the fundamental stretches for the HCHO (e.g., the symmetric C=O, out-of-plan bend, CH anti-symmetric stretch and CH_2_ rocking) [40,41,42,43]. Unlike its derivative, **TPN1-18** displays a simpler spectrum containing the signature absorptions for EtOH at 1256, 1040 and 978 cm^−1^, which represent the OH bend/CH_2_ twist, asymmetric and symmetric CCO stretches [38,39]. Both samples reveal similar characteristic IR absorptions for MeOH and EtOH which were observed previously for FRs containing the same phosphonates groups P(O)(OCH_3_)_2_ and P(O)(OC_2_H_5_)_2_ [35,44]. The particular interest is the stretch at 1533 cm^−1^ in **TPN3-20**. An earlier study indicates that the symmetric C-H bend in formaldehyde (HCHO) appears at around this frequency with the intensity of less than that of the C=O [42]. On the other hand, this frequency also corresponds to the absorption of the CH_3_ s-deformation of MeOH [39]. Thus, it is likely that the co-existence of both functional groups is accountable for the high intensity of this stretch. Further examination of **TPN1-18** and **TPN3-20** spectra finds that the asymmetric and symmetric absorptions for CH_2_ and CH_3_ appear around 3000 cm^−1^ but their vibrational signals are quite different. Overall, the first stage of degradation process seems to last longer for **TPN1-18** than **TPN3-20**. 

Among the three profiles, it is clear that without the FRs, more materials from the cotton fabric are degraded and that this process occurs at later time. Generally, the FTIR data is consistent with the TGA data.

#### 2.2.3. Information on Char: Functional Groups Observed by ATR-IR

**Figure 5:**
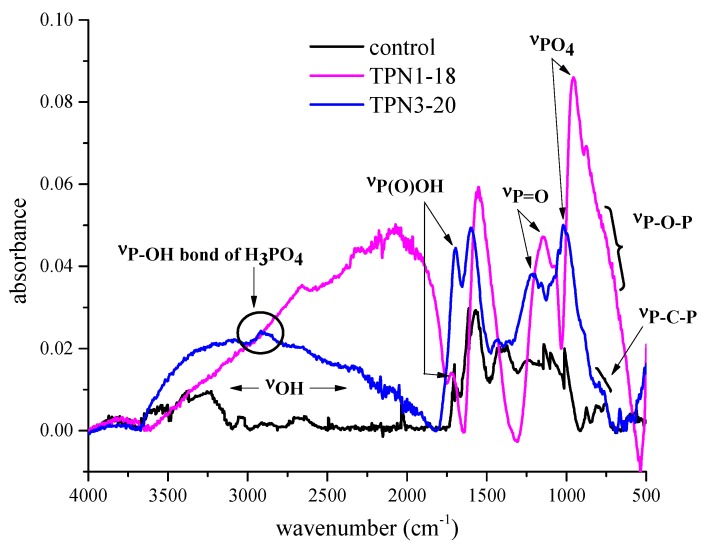
ATR-IR spectra of the burned control and treated fabrics.

To learn what was remained on the surface of the burned samples after the TGA-FTIR experiment, these samples were examined by ATR-IR and their spectral results are presented in Figure 5. All three samples show strong absorptions in the regions of 3500 and 1700–500 cm^−1^. For the control, the presence of aromatic-type structure recognized by the presence of the =C-H stretching vibrations and the ring breathing vibrations appear at around 3030 and 1600–1500 cm^−1^, respectively. Furthermore, the absorptions found in the region 1000–650 cm^−1^ may be due to the out of plane deformation vibrations of the ring [28]. These characteristic absorptions are in accordance with depolymerization of cellulose and formation of char [45].

Upon first inspection, both **TPN1-18** and **TPN3-20** spectra contain some common characteristics. One feature is the strong absorption at ~3000 cm^−1^, indicating the OH group. In this region, a stretch at around 2920 cm^−1^ in burned **TPN3-20** corresponds to the P-OH [46,47]. A closer look at the burned **TPN1-18** reveals a similar absorption at 2655 cm^−1^. In the lower frequency region, the strong absorptions at 1719 and 1693 cm^−1^ link to the P(O)OH functional group [28]. Furthermore, two stretches at 1154 and 1147 cm^−1^ arise from the stretching mode of the P=O [30,48]. In some cases, these stretches come from carbons activated with phosphoric acid [49,50]. The PO_4_^3−^ content in both samples gives strong evidence through the symmetric stretches for the P-O groups at 987 and 966 cm^−1^ [30].

Investigation of the fingerprint region of these spectra finds a few noticeable differences. The spectrum of **TPN1-18** displays a shoulder at 800–680 cm^−1^ which originates from the P-O-P group [30,51]. At 835–767 cm^−1^, **TPN3-20** reveals the absorptions for the P-C-P [52,53]. It is found that methylene diphosphonic acid gives distinct vibrational signals in this region, which does not appear in the spectra of higher homologues.

#### 2.2.4. ^31^P solid State NMR of Treated Fabrics

Further exploration of the unburned and burned surfaces of the treated fabrics using ^31^P solid state NMR experiment reveals structural components of the products in the solid phase. Four spectra corresponding with the data of the treated fabrics collected before and after the TGA-FTIR experiment are presented in Figure 5. In the unburned samples, ^31^P chemical shift of **TPN1-18** resonates at −1.7 ppm, while that of **TPN3-20** appears at 23.2 ppm. In both samples, the broader smaller peaks could, in theory, be due to hydrolysis products formed during the treatment process.

**Figure 6:**
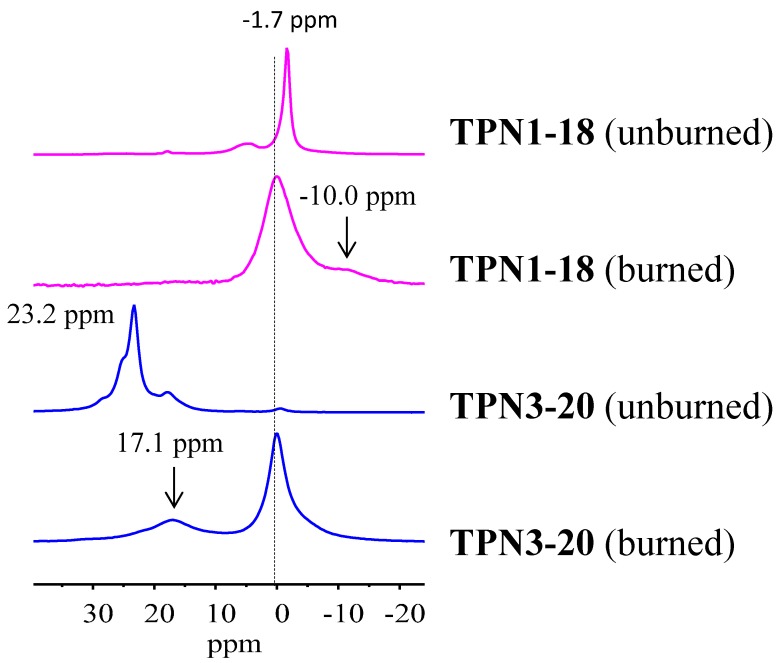
^31^P-NMR of **TPN1** and **TPN3** on the unburned and burned treated fabrics.

**Figure 7:**
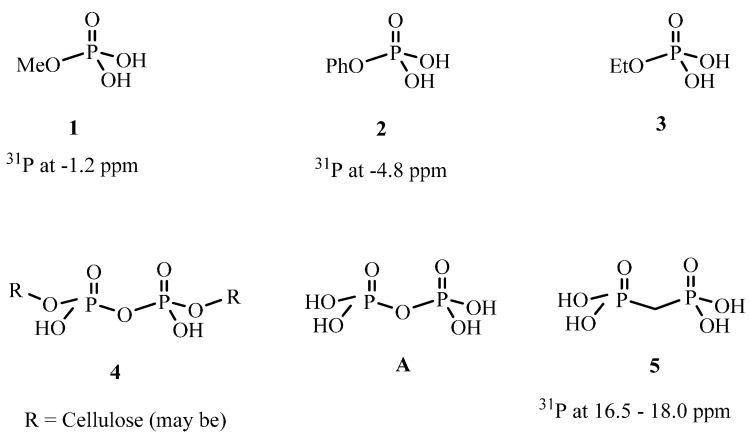
Compounds **1**, **2**, **3**, **4**, **A** and **5** [54,55,57,58].

As seen in Figure 6, upon burning **TPN1** and **TPN3** are converted to a same substance, which has a chemical shift at 0.0 ppm. It is known that the chemical shifts of mono-phosphorus for all non-cyclic, except that for tri-*t*-butyl and other tertiary alkyl derivative systems, are in a very narrow range of about −5 to +5 ppm [54]. Of particular interest is the chemical shift of H_3_PO_4_, at 0.0 ppm [55]. It has been observed that the solid state ^31^P-NMR spectra of a burned sample which originally contains a P(O)(OC_2_H_5_)_2_ group also exhibits a peak at around 0.0 ppm [56]. The authors consider that this burned sample may also contain phosphorus-acid species (*i.e*., species with a POH group in the structure). It is found that the chemical shifts of the derivatives of phosphoric acid are close to the acid’s value (e.g., **1** and **2**, Figure 7, have chemical shifts of −1.2 ppm [57] and −4.8 ppm [58], respectively). Based on the chemical shifts of **1** and **2**, the chemical shift of **3** is expected to be within this region as well. Thus, the major peak in the burned **TPN1-18** and **TPN3-20** spectra can neither be **1** nor **3** and is very likely H_3_PO_4_.

In addition to the H_3_PO_4_ peak, the burned spectra contain small peaks that appear at −10.0 ppm for **TPN1-18** and 17.1 ppm for **TPN3-20**. They are likely the side products from the decomposition of **TPN1** and **TPN3** with possible structures of **4** [59,60,61] and **5** [62,63,64] (Figure 7), respectively. Furthermore, the side product in **TPN1** cannot be the pyrophosphoric acid **A** (Figure 7) as this has a ^31^P chemical shift at −14 ppm [65]. It is also indicated that an acid like **A** has a phosphorus signal at −10.0 ppm when 5′-*O*-Nucleoside or 3′-*O*-Nucleoside replaces one OH on each side [59]. Thus, in this process it is speculated that the cellulose plays the same role as the two nucleosides. Interestingly, the partly burned sample of the compound that contains the P(O)(OC_2_H_5_)_2_ group mentioned above [56] also gives a peak at around 17–18 ppm in ^31^P-NMR spectrum. More importantly, this compound also contains -CH_2_-O- next to the phosphorus as in **TPN3**. The only difference is that the phosphorus in **TPN3** connects to OCH_3_ where as the phosphorus in that compound attaches to OC_2_H_5_. Unfortunately, no identification of the peak at 17–18 ppm is provided in the paper [56].

### 2.3. Predicted Energy Required Fragmenting **TPN1** and **TPN3** at Different Positions

**Figure 8:**
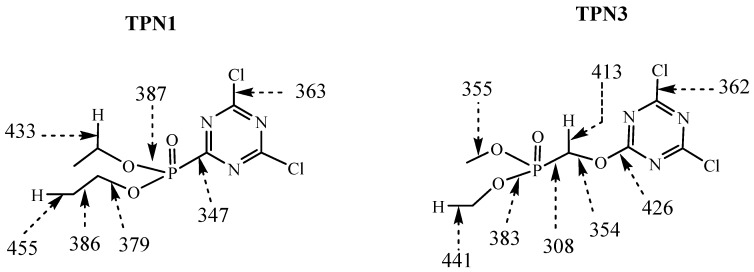
Bond dissociation energies (in kJ/mol) of **TPN1** and **TPN3**.

Thermal decomposition of a compound is often triggered by dissociation of the weakest bond and this establishes the general character of the later degradation pathways. The investigation of bond dissociation energy helps explain why one bond cleaves in preference to another. In general, the smaller the dissociation energy a bond has, the less stable that bond becomes. The degradation mechanism of **TPN1** and **TPN3** and the temperatures at which it occurs will depend mainly on their structures. Figure 8 summarizes the outcome of the computational studies on the selected bonds. The results provide some suggestions as to which bonds will be broken first and what type of products may be generated during thermal degradation. The studies on triazine ring are not included as the breaking aromatic rings normally results in much higher energy than other bonds [66,67,68].

As seen in Figure 8, the calculations show that the C-P bond is the weakest bond in comparison to the rest of the bonds in each system and the energy value is smaller in **TPN3** than it is in **TPN1**. This may be due to the connection of the C with other group; this means that C belongs to aromatic ring in **TPN1** but it is part of methylene group in **TPN3**. An investigation of BDE of organophosphorus compounds using high-level ab initio methods in different procedures BMK, M05-2X and SCS-ROMP2 also reveal that P-CH_3_ bond has lower BDE as compared to P-Ph bond [69]. In both structures the average bond is the C-Cl bond and the energy value for their bond dissociation is almost the same. Previous studies on BDE for this type of bond (*i.e*., N=C(Cl)-C or N=C(Cl)-N on mono- or di-substituted chloro-triazine, -pyrimidine, -pyrazine, and -pyridazine systems) show similar results [70].

### 2.4. Plausible Explanation for Thermal Degradation of **TPN1** and **TPN3**

Thermal degradation mechanisms are typically proposed after the investigation of the degradation products. Often, there is still significant discussion over the detailed path taken to form the final species. The data from all experiments for the control and treated fabrics and computational studies on **TPN1** and **TPN3** provide important evidence about the process.

**Scheme 1:**
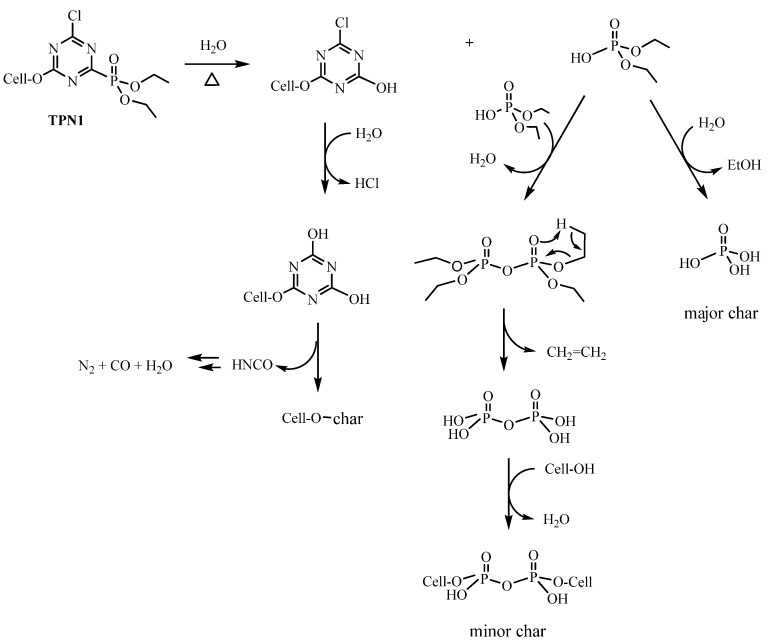
Plausible mechanism of thermal degradation of **TPN1**.

**Scheme 2:**
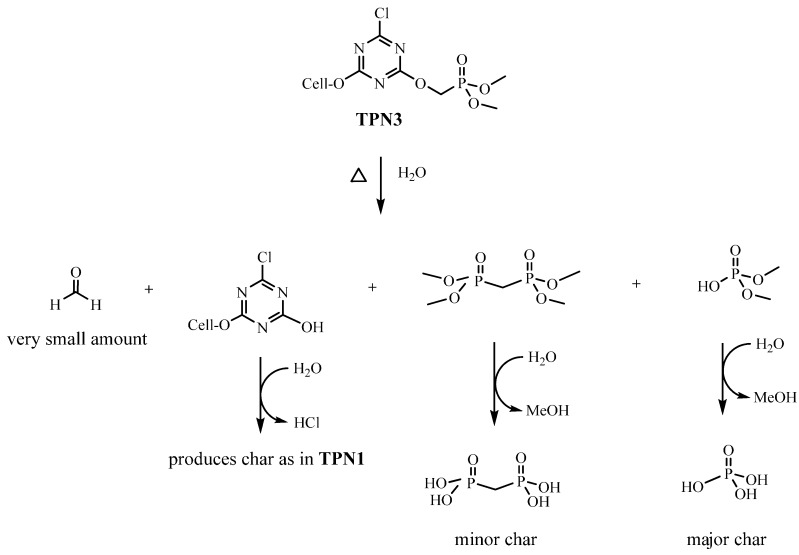
Plausible mechanism of thermal degradation of **TPN3**.

In the control, thermal decomposition leads to the depolymerization of the cellulose polymer to release various anhydrosugar derivavtives. Among them, the most common product is levoglucosan, which has been found to be a chemical tracer for cellulosic pyrolysis [44]. Unlike the control, this process is altered in the treated fabrics by the presence of FRs due to their catalytic action (Scheme 1 and Scheme 2). As seen in Figure 3b,c, it is obvious that more water and less gaseous products are produced from the breakdown of cellulose.

It is possible that water could play a crucial role in the thermal degradation of both compounds. The absorptions of P-OH in the char suggest that water (absorbed in cotton cellulose) could react with them at high temperature to form an acidic substance. Based on this evidence, **TPN1** and **TPN3** may first decompose by hydrolysis or elimination to release the acidic derivatives and triazine ring. These acidic derivatives may catalyze the formation of char, which is H_3_PO_4_. Further activities in both treated fabrics can be described as, in the case of **TPN1-18**, two phosphate esters (in small amount) combine to form pyrophosphonate derivative and to free H_2_O. The phosphonate moiety of the pyrophosphonate might further decompose to give up CH_2_=CH_2_ and to produce pyrophosphoric acid. This acid then phosphorylates the cellulose to form the minor char.

**Figure 9:**
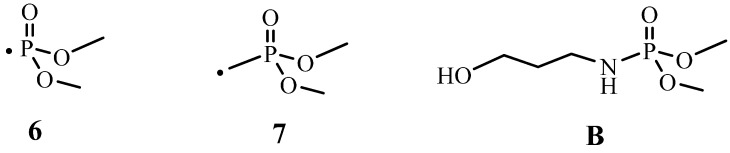
Species **6**, **7** and compound **B**.

On the other hand, the degradation of **TPN3-20** is more perplexing due to some side reactions. After the initial cleavage of the phosphonate group at the C-P bond, the CH_2_-O bond might be broken to generate a methylene species that might be reactive. This species then reacts with some phosphonate **6** (Figure 9), which comes from the initial breaking to form **7**. Later, **6** and **7** combine to give the methylene diphosphonic acid, a minor char. The computational studies also support the cleavage of the CH_2_-O bond as the next step. Moreover, the formation of some methylene species in the presence of water might produce the HCHO. This may account for the late appearance and small intensity in the C=O absorption of HCHO (Figure 4b). It is unlikely that the C-Cl bond not the CH_2_-O bond breaks after the breaking of C-P bond to give the HCHO since this step does not support the formation of methylene diphosphonic acid. One might reason that the MeOH released from the decomposition of **TPN3** can form the HCHO. Previous studies done by the same authors show that a phosphoramidate flame retardant **B** (Figure 9) containing the same phosphonate moiety does not produce HCHO when it decomposes [44]. It is unlikely, therefore, that the formation of HCHO in this experiment comes from the phosphonate moiety. Furthermore, the reaction requires much higher temperature for the MeOH to convert to the HCHO. As reasoned above, the dissociation of the phosphonate moieties results in the formation of two types of char in which the major char develops first. The formation of the major char causes a release of reasonable amount of MeOH and EtOH. In Figure 4a,b, the observable absorptions for the CH_2_ and CH_3_ at around 3000 cm^−1^ in MeOH and EtOH are only seen at a later time. It is likely that in their earlier stage these absorptions are masked by the release of the HCl. After the decomposition of the phosphonates in **TPN1** and **TPN3**, the cellulose starts to decompose. Since the H_3_PO_4_ layer acts as a thermal coating, it prevents the degradation of cellulose during this process.

In both of the cases, the fate of the triazine ring might be similar. The triazine ring forms covalent bond with the cellulose unit during the treatment based on the evidences of ATR-IR spectra and the difference in chemical shifts of the phosphorus of the compounds and the treated fabrics. Upon the detachment with the phosphonate and then the cleavage of the Cl, the ring forms cyanuric acid derivative. At temperatures greater than 300 °C, cyanuric acid degrades to yield the HNCO [71,72,73]. In the absence of O_2_, the newly formed HNCO is further broken down through a series of steps to produce N_2_, CO and water. In this experiment, the decomposition of HNCO is improbable to observe nevertheless because of the concurrence of the degradation of the fabrics (Figure 4a,b). 

The mode of action of **TPN1** and **TPN3** proposed in Scheme 1 and Scheme 2 takes place under a slow heating rate of 10 °C/min. With this heating rate, a necessary high degree of resolution can be obtained when a transition is encountered (*i.e*., 140 °C in TPN1-18 and 136 °C in TPN3-20 in Figure 3, TGA curves). Fire phenomena, which control what decomposition products may be, often happen at faster heating rates. In such cases, the decomposition stages of the two compounds may be reduced which result in the early formation of the final products [74,75]. Furthermore, the methylene diphosphonic acid (minor char) (Scheme 2) could be detectable at the end of the experiment, in the solid phase, but the HCHO might not be observable due to small amount formed during the experiment, in the gas phase.

## 3. Experimental Section

### 3.1. Materials

All reactions were conducted under nitrogen and were monitored by silica gel 60 F_254_ thin layer chromatography (TLC) from EMD (Damstadt, Germany). All purchased chemicals for the experiments came from Aldrich (St. Louis, MO, USA) and were used in their original forms. Twill fabric, style 423, with the weight of 258 g/m^2^ was obtained from Testfabrics, Inc. (West Pittston, PA, USA) as 100% cotton cellulose. This fabric was desized (starches removed), bleached and was cleared of all resins and finishes.

### 3.2. Synthesis

**Scheme 3:**
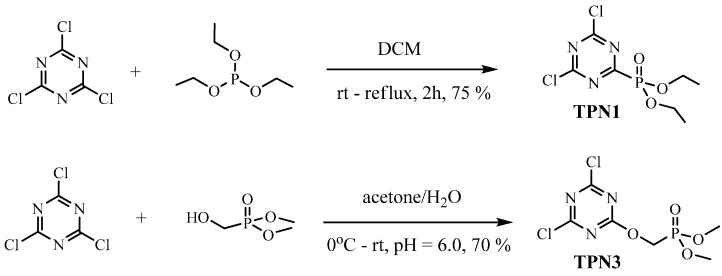
Synthesis of TPN1 and TPN3.

Scheme 3 shows the preparation of the two compounds **TPN1** and **TPN3**.

#### 3.2.1. Synthesis of **TPN1**

The preparation of **TPN1** was achieved following the published literature of Mikroyannidis [31]. A mixture of cyanuric chloride (5.0 g, 27.1 mmol) and dichloromethane (DCM) (50 mL) was stirred at room temperature until completely dissolved. Triethylphosphite (5.04 g, 27.7 mmol) in dry DCM (50 mL) was added dropwise to this solution. When the addition was complete, the reaction mixture was refluxed under nitrogen for 2 h and then monitored by thin layer chromatography (TLC) using 5% MeOH/DCM as an eluent. When the reaction was complete, the organic layer was separated, dried in vacuo and the residue was purified by column chromatography (c/c) (silica gel, 5% MeOH/DCM) to furnish **TPN1** as a yellowish oil in 75% yield. ^1^H-NMR (400-MHz, CDCl_3_) δ-ppm: 1.44–1.48 (m, 6H), 4.42 (t, 4H). ^13^C-NMR (100-MHz, CDCl_3_) δ-ppm: 16.4 (d, *J =* 6 Hz), 65.5 (d, *J =* 6 Hz), 172.4 (d, *J =* 20 Hz), 174.1–176.7 (d, *J =* 260 Hz). ^31^P-NMR (162-MHz, CDCl_3_) δ-ppm: 0.68. *m*/*z* observed 286.0 (calcd. 286.05).

#### 3.2.2. Synthesis of **TPN3**

This compound was synthesized according to the procedure established by Nguyen *et al.* [22]. In this reaction, **TPN3** was formed by a coupling reaction of dimethyl hydroxymethyl phosphonate with cyanuric chloride at around pH 6. After work-up, a yellowish oil was obtained. The crude was further purified by c/c to give the desired product as an off white solid in 70% yield. ^1^H-NMR (400-MHz, CDCl_3_) δ-ppm: 3.86 (d, 6H, *J =* 8), 4.83 (d, 2H, *J =* 8). ^13^C-NMR (100-MHz, CDCl_3_) δ-ppm: 53.8 (d, *J =* 6 Hz), 60.6 (d, *J =* 167 Hz), 170.9 (d, *J =* 8 Hz), 173.1 (s). ^31^P-NMR (162-MHz, CDCl_3_) δ-ppm: 18.8. *m*/*z* observed 287.97 (calcd. 288.03).

### 3.3. Fabric Treatment

Samples of twill fabric were soaked in 20% sodium hydroxide solution for two hours and padded at 40 psi using a padder (Birch Brothers Southern, Inc., Waxhaw, NC, USA). A required quantity of each chemical was dissolved in a minimum quantity of 50% aqueous isopropanol (*w*/*v*). The mercerized twill fabrics were soaked in these solutions for one hour and were padded at 10 psi (or 0.689 bar) to achieve approximately 110%–120% wet pickup. After that, the padded fabrics were mounted on pin frames, dried at 100 °C for 5 min and cured at 120 °C for 10 min in ovens (types LTF 146491 and LTE 18795, respectively, Mathis U.S.A., Inc., Concord, NC, USA). The fabrics were then rinse with deionized water for 10 min, tumble dried and allowed to cool to room temperature.

To obtain the wet pick-up and percent add-on for the fabrics, the weights before treatment (*w_a_*), after padding (*w_ap_*) and after cooling (*w_b_*) were fitted to Equations (1) and (2):

Wet pick-up (%) = [(*w_ap_− w_b_*)/*w_b_*] × 100 (1)

Add-on (%) = [(*w_a_− w_b_*)/*w_b_*] × 100 (2)

### 3.4. Instrumentation

#### 3.4.1. ATR-IR and TGA/FTIR

The functional groups within **TPN1** and **TPN3** molecules and cotton fabrics were identified by an A220/D01 Platinum Alpha ATR-IR spectrometer (Bruker Optics Inc., The Woodlands, TX, USA) equipped with a diamond crystal plate. These experiments were set to collect 42 interferograms at a resolution of 4 cm^−1^ in the range of 4000–600 cm^−1^. All data were analyzed by the Opus software (Opus 6.5, Licensed to Vertex 70/80 System of Bruker’s IR spectrometer, Billerica, MA, USA) that measures the intensity of the absorption band (representing the functional groups) as a function of temperature. The original images of all spectra were reconstructed by OriginLab 9 software (OriginPro 9.0.0 (32 bit) SR1, OriginLab Corporation, Northampton, MA, USA).

The TGA-FTIR experiment was conducted by a Q500 thermogravimetric analyzer (TA Instruments, New Castle, DE, USA) and a Tensor-27 spectrometer (Bruker Optics Inc.). In this experiment, –8.0 ± 0.2 mg of each sample of control and **TPN1-18** and **TPN3-20** was heated between 20 and 550 °C in the thermogravimetric analyzer at a rate of 10 °C/min and under a nitrogen flow rate of 90 mL/min. This had resulted volatile decomposition products which then traveled through a transfer line to reach the gas cell of the FTIR spectrometer. At the gas cell, they were analyzed by a liquid-nitrogen cooled MCT detector equipped with ZnSe window. The gas components were then recorded as the absorptions in the 4000–600 cm^−^^1^ region at a resolution of 4 cm^−1^. To maintain the gas state of these components both the transfer line and the gas cells had been kept at 200 °C. The FTIR data was obtained at every 5-degree increment along TGA heating profile or a 30 s delay between the timed measurements for the FTIR. All data analyses and image reconstructions were treated in the same way as in the ATR-IR experiment. 

#### 3.4.2. ^31^P Solid State NMR

This experiment was carried out on unburned and burned samples of treated fabrics **TPN1-18** and **TPN3-20**. Initially, all fabrics were ground by a Thosmas Wiley mill to produce 40-mesh samples. This study utilized a Bruker Avance 500 MHz (11.7 T) wide-bore spectrometer (Bruker, Billerica, MA, USA) equipped with a doubly-tuned magic angle spinning (MAS) probe. Each sample in this experiment was packed into a 4 mm zirconia rotor and spun at an MAS speed of 12 kHz. The NMR spectra were collected using high power proton decoupling during the acquisition period and signal averaged for either 1024 or 4096 transients. Spectra were externally referenced to a sample of 85% H_3_PO_4_ at 0.0 ppm.

### 3.5. BDE

The bond strength between triazine and two substituents chloride and phosphonate was obtained by calculating the DBE, defined here as the enthalpy change of the homolytic bond dissociation reaction R-Y → R^•^ + Y^•^. The calculations were performed by using density functional theory (DFT) procedure at B3LYP level run with a 6-31+G* or 6-31+G(d) basis set of Spartan 14 v1.1.4 package (Wavefunction, Inc., Irvine, CA, USA). This method was selected to accomplish the highest accuracy with a reasonable computational time.

## 4. Conclusions

By utilizing ATR-IR, TGA-FTIR and ^31^P solid state NMR techniques and the computational studies on BDE, extensive experiments were performed to investigate the mechanism of the thermal degradation for the control cotton fabric and the cotton fabrics treated with flame retardants **TPN1** and **TPN3**. The results indicate that: the control produces more flammable gas products which evolve at a later time; these gases arise from the depolymerization of cotton cellulose; the released gases observed for **TPN3-20** and **TPN1-18** originate from the decomposition of the FRs and cotton cellulose; the amount of gas products from cellulose appear to be lower for treated fabrics in reference to the control; more activities occur for the decomposition of **TPN3-20** than **TPN1-18**; and both chemicals yield phosphoric acid as a major char.
